# Long-term outcome of biopsy-proven idiopathic tubulointersitial nephritis with or without uveitis in children—a nationwide follow-up study

**DOI:** 10.1007/s00467-021-05060-5

**Published:** 2021-05-18

**Authors:** Sari Rytkönen, Juuso Tainio, Ville Saarela, Kira Endén, Janne Kataja, Pekka Arikoski, Matti Nuutinen, Timo Jahnukainen

**Affiliations:** 1 Department of Children and Adolescents and PEDEGO Research Unit, Oulu University, Oulu, Finland; 2 Department of Children and Adolescents, Oulu University Hospital, Oulu, Finland; 3 Department of Paediatric Nephrology and Transplantation, New Children’s Hospital, University of Helsinki and Helsinki University Hospital, Box 347, Stenbäckinkatu 9, 00029 Helsinki, HUS Finland; 4 Department of Ophthalmology, Oulu University Hospital, Oulu, Finland; 5 Department of Paediatrics, Tampere University Hospital, Tampere, Finland; 6 Department of Paediatrics and Adolescent Medicine, Turku University Hospital, Turku, Finland; 7 Department of Paediatrics, University of Eastern Finland and Kuopio University Hospital, Kuopio, Finland

**Keywords:** TIN, TINU, Child, Biopsy, Uveitis, Outcome, Stage 5 chronic kidney disease

## Abstract

**Background:**

Only a few studies reporting the long-term outcome of children with idiopathic tubulointerstitial nephritis (TIN) and uveitis syndrome (TINU) are available. We studied the long-term kidney and ocular outcome in a nationwide cohort of children with TIN or TINU.

**Methods:**

All patients followed up for a minimum of 1 year by a paediatrician and an ophthalmologist were enrolled. The data on plasma creatinine (P-Cr), estimated glomerular filtration rate (eGFR), proteinuria, hypertension and uveitis were collected retrospectively.

**Results:**

Fifty-two patients were studied. Median age at time of diagnosis was 13.1 (1.8–16.9) years and median follow-up time was 5.7 (1.1–21.2) years. Forty-five (87%) patients were initially treated with glucocorticoids. The median of the maximum P-Cr was 162 μmol/l (47–1,016) and that of eGFR 47 ml/min/1.73m^2^ (8–124). Uveitis was diagnosed in 33 patients (63%) and 21 (40%) patients developed chronic uveitis. P-Cr normalised in a median of 2 months. Eleven (21%) patients had nephritis recurrence during or after discontinuation of glucocorticoids. At the latest follow-up, 13 (25%) patients had eGFR < 90 ml/min/1.73m^2^ (median 83; 61–89 ml/min/1.73m^2^). Six patients had tubular proteinuria; all presented with TIN without uveitis. Seven (13%) patients were hypertensive. Eleven (21%) patients had uveitis. One patient developed uraemia and was later transplanted.

**Conclusions:**

Our study questions the previously reported good long-term kidney and ocular outcome of patients with TIN/TINU. Decreased kidney function and/or ocular co-morbidities may persist for several years; thus, both kidney and ocular follow-up for at least 1 year is warranted.

**Graphical abstract:**

A higher resolution version of the Graphical abstract is available as Supplementary information 
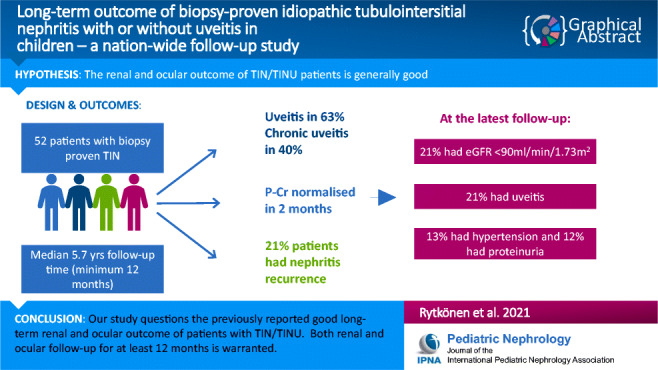

**Supplementary Information:**

The online version contains supplementary material available at 10.1007/s00467-021-05060-5.

## Introduction

Tubulointersitial nephritis (TIN) is an inflammatory disease affecting primarily the renal interstitium and tubular wall without significant glomerular or vascular involvement [1]. TIN can sometimes be accompanied by uveal inflammation, in which case it is referred to as tubulointerstitial nephritis and uveitis (TINU syndrome). There are previous data showing that uveitis may present as a chronic and/or relapsing form of the disease [1–4].

TIN can be triggered by several causes including infections and medications, such as antibiotics, nonsteroidal anti-inflammatory drugs and proton pump inhibitors, or the aetiology can be idiopathic [5]. Separately, TIN and uveitis can also be associated with systemic immunologic conditions such as sarcoidosis, systemic lupus erythematosus or inflammatory bowel disease [6–8]. TIN is a rare but significant cause of acute kidney insufficiency in children, accounting for approximately 7% of acute kidney injuries (AKI) in the paediatric population [9]. It is usually considered a condition with good long-term prognosis. However, there is evidence that some patients develop permanent kidney impairment, but studies reporting long-term outcomes of TIN/TINU syndrome in the paediatric population are scarce and follow-up times have been short [2, 4, 10–12]. Our aim was to evaluate the long-term kidney and ocular outcome in a nationwide cohort of children with idiopathic biopsy-proven TIN or TINU who were followed up by a paediatrician and an ophthalmologist according to a systematic follow-up protocol for at least 1 year.

## Methods

This study was part of our previous nationwide studies and detailed patient demographics have been reported before [3, 4, 13–15]. The study cohort was collected from all five university hospitals in Finland between 1995 and 2015. All patients with biopsy-proven TIN diagnosed before the age of 17 years were enrolled.

The diagnostic inclusion criteria of our study, whether or not the patient had uveitis, were tubulointerstitial changes found in the kidney biopsy. Criteria for kidney biopsy were typical presenting symptoms and clinical signs of TIN (Table 1), marked low molecular weight proteinuria (LMWP); either urinary alpha-1-microglobulin (U-α1-MG) or beta-2-microglobulin (U-β2-MG) and decreased eGFR. In all patients, diagnostic biopsy was performed before any immunomodulatory treatment was given. Meticulous work-up was done to exclude possible underlying conditions, such as drug-induced TIN, respiratory infection, sarcoidosis, connective tissue disorder and lymphoma. In order to find and exclude patients with secondary TIN, screening for respiratory viruses was performed by using viral serology, antigen detection and/or PCRs. Antinuclear antibodies, anti-neutrophilic cytoplasmic antibodies and tests for epidemic nephropathy (Puumala virus) were assayed. Drug-induced TIN was excluded by careful patient history. Complement components 3 and 4 and angiotensin-converting enzyme, and lysozyme concentrations were measured. In addition, sarcoidosis, connective tissue disorder and lymphomas were excluded by relevant X-ray and laboratory tests decided individually. The differential diagnoses setup was re-evaluated if TIN recurrence occurred. Kidney biopsy samples were re-evaluated from 26 patients and the findings were scored semiquantitatively and scores for tubulointerstitial activity and chronicity indexes were calculated as follows: tubulitis (0–3), interstitial inflammation (0–3), tubular atrophy (0–3) and interstitial fibrosis (0–3). From the remaining 26 patients, the scores were collected from the pathologist statements recorded on the patient files.

**Table 1:** Clinical signs and symptoms before the TIN diagnosis and the findings of the diagnostic kidney biopsy. The data are presented separately for all patients, TIN, TINU and TINU with chronic uveitis

Feature	All patients *n* = 52	TIN *n* = 19	TINU *n* = 33	*p* value^f^	TINU with chronic uveitis *n* = 21	*p* value^†^
General symptoms						
Fever	39 (75)	15 (83)	24 (73)	0.50^#^	16 (75)	0.97
Fatigue	41 (79)	13 (72)	28 (85)	0.30^#^	18 (86)	0.42^#^
Headache	18 (35)	6 (33)	12 (36)	0.40	9 (43)	0.48^#^
Anorexia	21 (40)	11 (61)	10 (30)	0.04	8 (38)	0.78
Arthralgia	11 (21)	3 (17)	8 (24)	0.73	5 (24)	0.75^#^
Weight loss	24 (46)	8 (44)	16 (49)	0.78	11 (52)	0.58
Cough	15 (29)	6 (33)	9 (27)	0.75	7 (33)	0.76
Gastrointestinal symptoms						
Abdominal pain	16 (31)	9 (50)	7 (21)	0.06	3 (14)	0.04
Vomiting	9 (17)	6 (33)	3 (9)	0.03^#^	2 (10)	0.20
Urinary findings and symptoms						
Polyuria	6 12)	0 (0)	6 (18)	0.05^#^	5 (24)	0.02^#^
LMWP*	50 (100)	19 (100)	31(100)	-	21 (100)	-
Glucosuria	48 (92)	17 (90)	31 (97)^ϕ^	0.28^#^	19 (95)*	0.83^#^
Pyuria (*n* = 49)	15 (29)	6 (35)*	9 (28)*	0.75	7 (35)*	0.75
Ocular symptoms	3 (6)	0 (0)	3 (9)	0.19^#^	2 (10)	0.36^#^
Dialysis	3 (6)	3 (16)	0	0.04	0	0.14
Biopsy findings						
Tubulitis	1 (0–3)	2 (0–3)	1 (0–2)		1 (0–2)	0.03
Interstitial inflammation	2 (0–3)	2 (1–3)	2 (0–3)	0.58	2 (1–3)	0.75
Interstitial fibrosis gr. 1	10 (19)	3 (16)	7 (21)	0.73	4 (19)	0.98
Tubular atrophy gr. 1	14 (27)	6 (32)	8 (24)	0.57	5 (24)	0.68

*TIN*, tubulointerstitial nephritis; *TINU*, tubulointerstitial nephritis with uveitis syndrome; chronic uveitis was defined as uveitis that had lasted more than 3 months despite treatment, with relapse within 3 months after discontinuing treatment. *LMWP*, low molecular weight proteinuria; treatment delay was defined as the time from onset of symptoms to the beginning of cortisone treatment

Percentage (range), χ^2^ –test for categorical variables was used, but if one cell or more had expected count less than 5 (#), we used Fisher’s exact test and exact Sig. (2-sided)

*Data missing in 1 patient

^ϕ^Data missing in 2 patients

*f p* values are for differences between TIN and all TINU patients

^†^*p* values are for differences between patients with chronic uveitis and all other patients (TIN and TINU without chronic uveitis)

A paediatric nephrologist and an ophthalmologist followed up all the patients. The follow-up protocol was comparable in all centres and included visits at least 1, 3, 6 and 12 months following the diagnosis, and at least annually thereafter if full remission was not achieved. The follow-up data were collected until June 30, 2020. Data on plasma creatinine (P-Cr), estimated GFR (eGFR), blood pressure, urine protein excretion and presence of uveitis were collected. Estimated GFR was determined according to the Schwartz formula [16]. At the follow-up visits, proteinuria was evaluated with a dipstick and in case of a positive result, with quantitative measurement of urinary albumin and low-molecular-weight (LMW) protein concentration (U-α1-MG or β2-MG). Persisting LMWP was considered an indicator of renal tubular dysfunction. The cut-off values were U-α1-MG/creatinine ratio > 2.2 mg/mmol or U-β2-MG/creatinine ratio > 35 ug/mmol. U-β2-MG concentration above 250 ug/ml or U-α1-MG concentrations above 6–12 mg/l depending on the subject’s age was considered abnormal. Hypertension was stated if blood pressure was above 95^th^ percentile according to gender, age and height or if the patient was on antihypertensive medication [17]. Information about the medical treatment for TIN/TINU and nephritis recurrences was collected from electronic patient records. Uveitis was classified according to standardisation of uveitis nomenclature (SUN) criteria [18]. Relapsing (recurrent) uveitis was thus defined as two uveitis episodes separated by a 3-month or longer period of inactivity without treatment in between. Chronic uveitis was defined in case of relapse within < 3 months after discontinuing treatment.

The same initial glucocorticoid treatment protocol was used at each centre [3]. Briefly, oral prednisone starting at a dose of 2 mg/kg/day (maximum 60 mg/d) was initiated after the diagnostic biopsy was performed. In the case of full kidney function recovery, the dose was reduced after 4-week treatment to 40 mg/m^2^ every other day (eod) for 1 week and tapered in 1-week steps by 10 mg/m^2^ eod during the next 3 weeks. In the case of partial kidney function recovery (LMWP and/or elevated P-Cr) at 1 month, the prednisone dose was reduced to 1.5 mg/kg eod for a 2-month period, after which tapered by 0.5 mg/kg eod in 1-month steps.

Statistical analyses were performed with IBM SPSS Statistics for Macintosh, version 25. Armonk, NY: IBM Corp. We used the Mann–Whitney *U* test to compare median values between two groups and the *χ*^2^ test or Fisher’s exact test, as appropriate, for comparing categorical variables. Descriptive data are presented as median values and range from the lowest to the highest value. Statistical significance was defined as *p* < 0.05.

## Results

### Patient demographics and baseline data

Altogether, 52 patients were enrolled over a 21-year study period. Twenty-nine (56%) patients were female. Demographic data and the key laboratory findings at the time of diagnostic biopsy are presented and at the latest follow-up visit in Table 2. The median age at diagnosis was 13.1 years (range 1.8–16.9 years) and the median follow-up time was 5.7 years (range 1.1–21.2 years). Uveitis was diagnosed in 33 patients (63%); 19 of them (58%) were girls. In most cases, uveitis was diagnosed at the same time with TIN, but in four (12%) patients, uveitis appeared 3–10 months after TIN. Twenty-one of the 33 uveitis patients (40%) developed chronic uveitis.

**Table 2:** Patient demographics and key laboratory findings at the time of the diagnostic kidney biopsy and the follow-up data. The data are presented separately for all patients, TIN, TINU and TINU with chronic uveitis

	All patients *n* = 52	TIN *n* = 19	TINU *n* = 33	*p* value^f^	TINU with chronic uveitis *n* = 21	*p* value^†^
Age at diagnosis, years	13.1 (1.8-16.9)	11.8 (1.8–15.4)	13.8 (5.6–16.9)	0.38	13.9 (5.6–16.9)	0.28
Median follow-up time, years	5.7 (1.1–21.2)	5.6 (1.1–15.5)	4.5 (1.2–21.2)	0.54	6.1 (1.9–21.2)	0.06
Age at the latest follow-up, years	18.4 (4.5–31.5)	16.7 (7.7–28.8)	17.9 (4.5–31.5)	0.21	18.5 (11.3–31.5)	0.03
Initial data						
CRP, mg/l (*n *= 41)	50 (3–224)	50 (3–224)*	43 (3–135)^#^	0.80	33 (3–110)^#^	0.77
ESR, mm/h	94 (9–140)	102 (64–128)*	86 (9–128)	0.68	79 (14–128)	0.07
P-Cr, μmol/l	162 (47–1016)	234 (64–1016)	139 (47–416)	0.01	169 (47–416)	0.24
P-Urea, mmol/l (*n* = 49)	9.0 (4.4–32.7)	10.8 (5.5–32.7)	7.9 (4.4–18.3)	0.04	8.5 (4.7–16.8)	0.28
U-αl-MG mg/l (*n* = 14)	80 (11–246)	141 (75–246)	78 (11–137)	0.40	76 (11–137)	0.52
U-β2-MG μg/l (*n* = 36)	24250 (1600–120000)	23438 (2090–12000)	24800 (1600–110200)	0.64	34000 (1600–110200)	0.33
Haemoglobin, g/L	103 (85–129)	99 (85–126)	104 (86–129)	0.14	104 (94–129)	0.30
WBC, E^9^/L (*n* = 39)	8.9 (4.3–31.0)	8.4 (5.9–31.0)	8.9 (4.3–12.9)	0.76	8.8 (4.3–12.9)	0.63
B-Eosinophiles, E^9^/l (*n* = 39)	0.31 (0–0.96)	0.31 (0–0.71)	0.31 (0–0.96)	0.90	0.35 (0–0.84)	0.67
P-Pi, mmol/l (*n* = 33)	1.09 (0.68–2.27)	0.98 (0.78–2.27)	1.11 (0.68–1.46)	0.35	1.09 (0.68–1.46)	0.80
Follow-up data						
P-Cr, μmol/l	72 (37–112)	64 (37–111)	74 (50–112)	0.52	76 (50–112)	0.28
eGFR, ml/min/1.73 m^2^	106 (61–183)	117 (61–183)	106 (68–158)	0.64	109 (68–158)	0.68
eGFR < 90 ml/min/1.73 m^2^, *n* (%)	13 (25)	5 (28)	8 (24)	0.78	5 (24)	0.82
CKD 5*, *n* (%)	1 (2)	1 (6)	0	-	0	-
Elevated blood pressure, *n* (%)	7 (13)	2 (11)	5 (15)	0.69	3 (14)	0.92
LMWP *n* (%)	6 (12)	6 (33)	0 (0)	-	0 (0)	-
Nephritic relapse	12 (23)	5 (26)	7 (21)	0.67	6 (29)	0.44

*TIN*, tubulointerstitial nephritis; *TINU*, tubulointerstitial nephritis with uveitis syndrome; chronic uveitis was defined as uveitis lasting more than 3 months despite treatment and/or with relapse within 3 months after discontinuing treatment. *CRP*, C-reactive protein; *ESR*, erythrocyte sedimentation rate; *P-Cr*, plasma creatinine concentration; *eGFR*, estimated glomerular filtration rate; *U-α1-MG*, urine alpha-1-microglobulin concentration (normal value < 15 mg/l); U*-β2-MG*, urine beta-2-microglobulin concentration (normal value < 250 μg/l); *WBC*, white blood cells; *P-Pi*, plasma phosphate concentration; *CKD 5*, stage 5 chronic kidney disease; reported values are median values with range in brackets. For continuous variables, the non-parametric Mann–Whitney *U* test was used. A two-tailed *p* value is reported

*TIN *n* = 13 for CRP and *n* = 17 for ESR

#TINU *n =* 28 and *n* = 19 in TINU with chronic uveitis group

*f* Median values were compared between TIN and TINU patients

†Median values were compared between patients with chronic uveitis and all other patients (TIN and TINU without chronic uveitis)

Fever, fatigue and weight loss were among the most common presenting symptoms in all patient groups (Table 1). Gastrointestinal symptoms, anorexia (61%), abdominal pain (50%) and vomiting (33%) were common as presenting clinical symptoms especially in TIN patients (Table 1). At the time of diagnosis, the median peak P-Cr was 162 μmol/l (range 47–1016) and eGFR 47 ml/min (8–124). Plasma phosphate (P-Pi) concentration was below the lower limit normal in 55% of the patients. In an additional five patients, P-Pi was within normal range at presentation, but decreased to hypophosphatemia after resolution of acute kidney injury. There was no difference in P-Pi concentrations between TIN and TINU patients (Table 2). Urinary Pi concentrations were not measured routinely. All patients presented with LMWP and the vast majority had glucosuria at the time of diagnosis. Pyuria was found in 16 (31%) of the patients. Eosinophils were not systematically investigated in urine samples (Table 1). The initial P-Cr was higher in the patients with isolated nephritis (234 μmol/l) than in the patients with TINU syndrome (139 μmol/l; *p* = 0.007) and eGFR was correspondingly lower in TIN patients (35 ml/min) than in patients with TINU (59 ml/min, *p* = 0.008) (Table 2). Patients with isolated TIN required dialysis more often than those diagnosed with concomitant uveitis (15.8% versus 0%, *p* = 0.04, respectively). The tubulitis score in the diagnostic kidney biopsy was significantly higher in TIN patients than in TINU patients (*p* = 0.001) or in patients with chronic uveitis (*p* = 0.03) (Table 1).

Forty-five (87%) of the patients were initially treated with prednisone. The median duration of therapy was 6 (0.1–66.0) months. In TINU patients, the treatment time was significantly shorter than in patients with TIN or chronic uveitis (Table 3).

**Table 3:** The treatment delay, frequency, length and efficiency of the glucocorticoid therapy and the number of nephritis relapses. The data are presented separately for patients with isolated tubulointerstitial nephritis, patients with uveitis and chronic uveitis

	TIN *n* = 19	TINU *n* = 12	TINU with chronic uveitis *n* = 21	*p* value
Corticosteroid therapy, *n* (%)	18 (100)	10 (83)	17 (81)	0.15
Treatment delay, days	31 (11–173)	41 (13–190)	38 (18–190)*	0.16
Duration of medication, months	7.5 (2–24)	2 (0.1–12)	6.5 (2–66) *n* = 15	0.01
Time to normal creatinine, months	1 (1–12)*	1 (0–3)^#^	2 (0–36)	0.06
Time to normal ESR, months	2 (1–6) *n* = 16	1 (0–6)^#^	1 (0–11)	0.67
Nephritis relapse, *n* (%)	5 (26)	1 (8)	6 (29)*	0.40

Data is presented as *n* (%) or median (range). Analyses performed by Chi-squared or Kruskal–Wallis test, as appropriate. A two-tailed *p* value is reported. Treatment delay was defined as the time from onset of symptoms to the beginning of cortisone treatment. *ESR*, erythrocyte sedimentation rate

*Missing two cases; # missing one case

### Kidney outcome

At the latest follow-up visit, the median P-Cr was 72 μmol/l (37–112) and median eGFR 106 ml/min/1.73 m^2^ (61–183) (Table 2). Thirteen patients (25%) had eGFR < 90 ml/min/1.73 m^2^ (median 83 ml/min/1.73 m^2^ (61–89)) but none of those had eGFR < 60 ml/min/1.73 m^2^. One additional patient underwent kidney transplantation 10 years after the TIN diagnosis. At the time of diagnosis, the median P-Cr tended to be higher in the patients with later reduced eGFR when compared to those with eGFR > 90 ml/min/1.73m^2^ at the last follow-up; however, the difference did not reach statistical significance (Table 4). The initial laboratory findings and biopsy scores were compared between the two groups and the only significant difference was higher interstitial inflammation score (*p* = 0.04) among patients with eGFR > 90 ml/min/1.73 m^2^ at the latest follow-up visit (Table 4).

**Table 4:** The key laboratory findings at the time of the diagnostic biopsy, clinical parameters and corticosteroid treatment time according to glomerular filtration rate below and above 90 ml/min/1.73 m^2^ at the latest follow-up

	eGFR < 90 ml/min/1.73 m^2^ *n* = 14	eGFR > 90 ml/min/1.73 m^2^ *n* = 38	*p* value
Age at diagnosis, years	13.8 (5.6–16.0)	12.6 (1.8–16.9)	0.10
P-Cr at diagnosis, μmol/l	192 (74–844)	153 (47–1016)	0.32
eGFR at diagnosis, ml/min/1.73 m^2^	55 (13–95)	46 (8–124)	0.81
ESR at diagnosis, mm/h	95 (17–114), (*n* = 10)	94 (9–140), (*n* = 35)	0.56
Haemoglobin, g/l	107 (86–126)	99 (85–129)	0.45
WBC, E^9^/l (*n* = 39)	7.6 (5.9–10.6)	8.9 (4.3–31.0)	0.20
Eosinophiles, E^9^/l (*n* = 39)	0.27 (0.06–0.54)	0.33 (0–0.69)	0.38
P-Pi, mmol/l (*n* = 33)	0.85 (0.68–1.49)	1.10 (0.78–2.27)	0.15
P-Urea, mmol/l (*n* = 49)	7.1 (4.4–24.8)	9.5 (4.7–32.7)	0.19
Glucosuria (*n* = 51)	13 (100)	35 (92)	0.30
Pyuria (*n* = 50)	3 (23)	13 (35)	0.43
Dialysis	0	3 (8)	0.28
TINU (%)	8 (57)	25 (64)	0.75
Chronic uveitis, *n* (%)	5 (33)	16 (42)	0.76
Treatment delay, days	44 (16-190)	35 (11-173)	0.67
Corticosteroid therapy, *n* (%)	13 (100)	33 (85)	0.12
Duration of medication, months	9.5 (0.1–15)	6 (1–48)	0.49
Time to normal creatinine, months	2.5 (1–14)	1 (0–36)	0.02
Nephritis relapse, *n* (%)	3 (23)	9 (21)	0.87
Interstitial inflammation	1 (0–3)	2 (1–3)	0.04
Interstitial fibrosis gr. 1	3 (21)	7 (18)	0.81
Tubular atrophy gr. 1	4 (29)	10 (26)	0.87

Data presented as *n* (%) or median (range) and analyses performed by Chi-squared or Kruskal–Wallis test, as appropriate. Two-tailed *p* value is reported. Number of subjects within group presented if missing cases. *P-Cr*, plasma creatinine; *eGFR*, estimated glomerular filtration rate; *ESR*, erythrocyte sedimentation rate; *WBC*, white blood cells; *P–Pi*, plasma phosphate concentration; *TINU*, tubulointerstitial nephritis and uveitis; treatment delay was defined as the time from onset of symptoms to the beginning of cortisone treatment

In our TIN/TINU cohort, P-Cr normalised at a median of 2 (1–36) months. In patients with final eGFR < 90 ml/min/1.73 m^2^, the median time to normal P-Cr was 2.5 months, which was significantly longer (*p* = 0.02) than in those with normal eGFR at the latest follow-up visit (Table 4). There was no statistically significant difference in the P-Cr normalisation rate between the patients with or without uveitis (Table 3). However, in patients with chronic uveitis, the normalisation time of P-Cr (median of two months; range 0–36 months) was statistically significantly (*p* = 0.021) longer than in TINU patients (1 month; range 0–3 months). Eleven patients had nephritis recurrence, defined as elevated P-Cr and increased urinary microglobulin excretion, during prednisone weaning. Recurrence was found in five TIN patients and five patients with chronic uveitis and in only one TINU patient. However, the recurrence rate did not differ statistically significantly between the groups (Table 3). Four patients with poor treatment response underwent a second biopsy due to prolonged nephritis symptoms. One patient was diagnosed with sarcoidosis and since excluded from the study. Two patients finally achieved remission. One patient did not achieve renal remission with glucocorticoid treatment and received additional azathioprine and cyclosporine A therapy for persistent TIN. He was still on cyclosporine monotherapy during his latest follow-up visit at the age of 22 years.

At the latest follow-up visit, only one patient had mild proteinuria (1+ in dipstick) in urine analysis. Urinary LMW protein excretion was elevated in 6 out of 36 patients analysed (17%); all six cases among the 18 TIN patients presenting without uveitis. Seven (13%) patients had elevated blood pressure and five (10%) were on antihypertensive medication. Four (57%) of these seven hypertensive patients had tubular atrophy and/or interstitial fibrosis in their diagnostic kidney biopsy, suggesting chronic changes. Two patients had high plasma renin concentration but no evidence of renal arterial stenosis. We did not find any statistically significant differences in eGFR, P-Cr or in the occurrence of hypertension in patients with or without uveitis at the latest follow-up visit.

### Ocular outcome

Eleven patients (21%) had uveitis at the latest follow-up visit (Table 1). Eight patients were on topical prednisone treatment at the latest follow-up. Two of them also had methotrexate treatment. One additional patient was on methotrexate monotherapy, and one had mycophenolate mofetil and adalimumab treatments for persistent uveitis.

One patient presented severe treatment-resistant uveitis. She was diagnosed with uveitis concomitantly with TIN. Her eGFR was 22 ml/min/1.73 m^2^ at the time of diagnosis. She was initially treated with intravenous methylprednisone pulses followed by oral prednisone, cyclosporine A, mycophenolate mofetil, infliximab and finally, adalimumab before ocular remission was achieved. Her eGFR was 112 ml/min/1.73 m^2^ at the last follow-up visit.

The initial laboratory parameters and biopsy findings did not differ significantly between those patients with chronic/recurrent uveitis and the others. The only exception was the tubulitis score in diagnostic kidney biopsy, which was higher in patients without chronic uveitis (Table 1).

## Discussion

To our knowledge, the present study describes the largest (52 children) cohort of biopsy-proven paediatric TIN patients with the longest follow-up time (5.7 years) reported in the literature [1, 2, 10, 11, 19, 20]. Unlike other studies, we only included patients with idiopathic TIN. Adult TIN follow-up cohorts are also few and small in size, and they also contain other than idiopathic TIN patients [21–23].

Our results question the general supposition that the outcome of paediatric patients with idiopathic TIN is generally good [2, 4, 12], since 25% of the patients had CKD stage 2 (eGFR 60–89 ml/min/1.73 m^2^) and one patient (2%) had developed stage 5 chronic kidney disease at the latest follow-up visit. Neither was the ocular outcome optimal, since uveitis was diagnosed in 63% of the patients and 21% of all studied patients had active uveitis after a median 68 months follow-up, which further highlights the importance of ocular follow-up in this patient group. LMWP was found in 17% of the patients; interestingly, all cases were among those with isolated TIN without uveitis. Unfortunately, we could not identify any clinical parameters or biopsy findings in our cohort which would predict long-term kidney outcome.

TIN is known to account for up to 25% of AKIs in the adult population and 5–7% in children [9, 24, 25]. Data regarding long-term kidney outcomes are scarce. In the adult population, subnormal kidney function was found in 50% of the TIN patients 18 months after the diagnosis [25]. In a recent study by Howell et al. [2], 60% of the paediatric patients with TIN had eGFR below 80 ml/min/1.73 m^2^ after 21 months follow-up. In the present study, kidney function was classified according to the KDIGO CKD categories. One-quarter of this cohort had eGFR < 90 ml/min/1.73 m^2^ at their latest follow-up visit. In 16% of the patients, eGFR was below 80 ml/min/1.73 m^2^. This is in accordance with our previous study reporting declined eGFR in 4/26 (15%) of the patients, the follow-up time being 33 months [4]. The better kidney outcome in our study may be explained by a longer follow-up time. In the present study, the median time to P-Cr normalisation was 2 months; however, the range was up to 36 months, suggesting that late recovery of kidney function is possible. This is also supported by Clarkson et al. in their study in an adult TIN population [24].

The role of glucocorticoid treatment in kidney outcome has remained unclear. There are some studies suggesting a favourable effect of prednisone treatment on kidney function in adults with drug-induced TIN [24, 25]. Our previous small prospective study suggested that prednisone accelerated kidney recovery in paediatric patients with idiopathic TIN; however, there was no significant difference in GFR 6 months after the diagnosis [26]. The present study is not able to clarify this issue, since 45 of the 52 patients (87%) had received prednisone treatment. However, it appears that prednisone has had a favourable effect on TIN, since nephritis relapse was found in 11 patients (21%) during prednisone weaning. It is also noticeable that the treatment time in patients with isolated TIN and patients with chronic TINU did not differ, suggesting that long-lasting oral glucocorticoid treatment was not entirely due to chronic uveitis.

Uveitis appears to be more frequent among paediatric TIN patients than in adults [2, 4, 27]. In the present study, 63% of the patients had TINU, which is in accordance with the findings by Howell et al. and Roy et al. [2, 11]. According to the literature, in about half of the cases uveitis was diagnosed without any ocular symptoms and simultaneously with TIN [2–4]. In our present cohort, 8% of uveitis cases were diagnosed several months after the occurrence of nephritis despite normal initial ocular findings. It is also important to note that 40% of our patients developed chronic uveitis and in 21%, uveitis was still active at the latest follow-up visit after a median of 6.1 years (range 1.9–21.2). The risk of persisting uveitis should be taken into account especially after weaning off glucocorticoids. In our current protocol, all paediatric patients with idiopathic TIN visit an ophthalmologist at the time of TIN diagnosis and three monthly for at least 1 year.

The pathomechanisms behind TIN have remained unclear. There are data suggesting that it is an autoimmune disorder with susceptibility to becoming chronic [14, 15]. In previous studies, we and others have shown that there are associations between certain human leukocyte antigen types [13, 28, 29], genetic variations in the inflammatory mediators [15], and that regulatory T cell function is altered [14] in patients with TIN/TINU syndrome and chronic uveitis, which may suggest autoimmune origin. This could, at least theoretically, increase the risk for chronic kidney dysfunction and kidney failure; however, there is some evidence that patients with the worst kidney outcome have had some other additional condition of autoimmune origin, such as sarcoidosis or inflammatory bowel disease [30–32]. Interestingly, in the present cohort, patients with isolated nephritis had significantly higher P-Cr at the time of the diagnostic biopsy. In addition, LMWP was identified at the latest follow-up visit in TIN patients only. On the other hand, there was no difference in the final eGFR between the patients with isolated TIN and TINU syndrome. The definite diagnosis of TIN is based on kidney biopsy and it is possible that, as in many other conditions, there are different conditions behind similar histological findings. This may in some cases lead to variation in the long-term kidney outcome.

Our study has some weaknesses. As in all retrospective studies, the available data are limited in the present study. However, in Finland, we have developed a uniform follow-up protocol for TIN/TINU patients. Therefore, the frequency and length of follow-up are quite similar in all paediatric nephrology centres in Finland. Another caveat is the relatively limited number of study subjects. However, TIN is a rare disease in the paediatric population and there are no previous paediatric data with a cohort this large (*n* = 52) and follow-up time this long (5.7 years), which can be considered a strength of our study.

In conclusion, we and others have previously shown that in most patients with acute idiopathic TIN, the recovery of kidney function is good. However, our study questions the good long-term kidney and ocular outcome of the patients with TIN/TINU. Decreased kidney function and/or ocular co-morbidities may persist for several years, and both kidney and ocular follow-up for at least 1 year is therefore warranted.

## Supplementary Information

ESM 1 (PPTX 45 kb)

## Data Availability

The datasets generated during and/or analysed during the current study are available from the corresponding author on reasonable request.
