# Supplementary material for: Long-term outcome of biopsy-proven idiopathic tubulointersitial nephritis with or without uveitis in children—a nationwide follow-up study
Source: Pediatr Nephrol. 2021 May 18;36(11):3663–71. doi: 10.1007/s00467-021-05060-5 (PMC8497450; doi:10.1007/s00467-021-05060-5)
Supplement: Supplementary file 1 — (PPTX 45 kb) [file 467_2021_5060_MOESM1_ESM.pptx]

## Slide 1
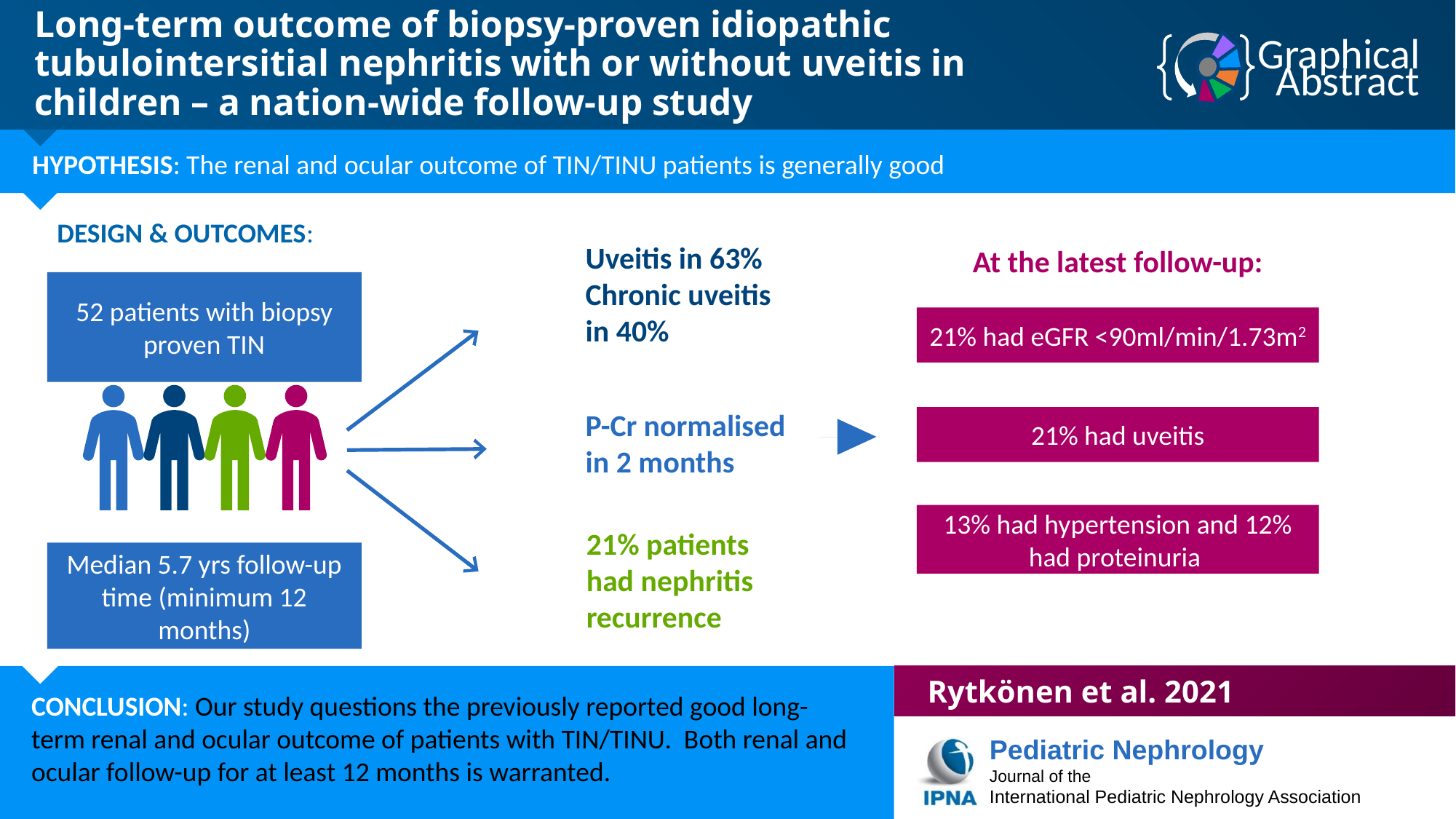

Long-term outcome of biopsy-proven idiopathic tubulointersitial nephritis with or without uveitis in
children – a nation-wide follow-up study
HYPOTHESIS: The renal and ocular outcome of TIN/TINU patients is generally good
DESIGN & OUTCOMES:
Uveitis in 63% Chronic uveitis in 40%
At the latest follow-up:
52 patients with biopsy proven TIN
21% had eGFR <90ml/min/1.73m2
P-Cr normalised in 2 months
21% had uveitis
13% had hypertension and 12% had proteinuria
21% patients had nephritis recurrence
Median 5.7 yrs follow-up time (minimum 12 months)
Rytkönen et al. 2021
CONCLUSION: Our study questions the previously reported good long-term renal and ocular outcome of patients with TIN/TINU. Both renal and ocular follow-up for at least 12 months is warranted.
